# Supplementary material for: The Synthesis of Sponge-like V2O5/CNT Hybrid Nanostructures Using Vertically Aligned CNTs as Templates
Source: Nanomaterials (Basel). 2024 Jan 18;14(2):211. doi: 10.3390/nano14020211 (PMC10820936; doi:10.3390/nano14020211)
Supplement: Supplementary file 1 [file nanomaterials-14-00211-s001.zip › nanomaterials-2807031-supplementary.pdf]

Supplementary Materials

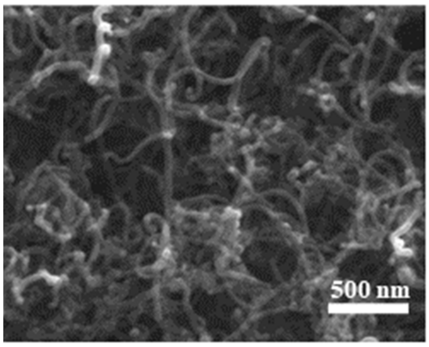

Figure S1. SEM images of VACNTs as grown.

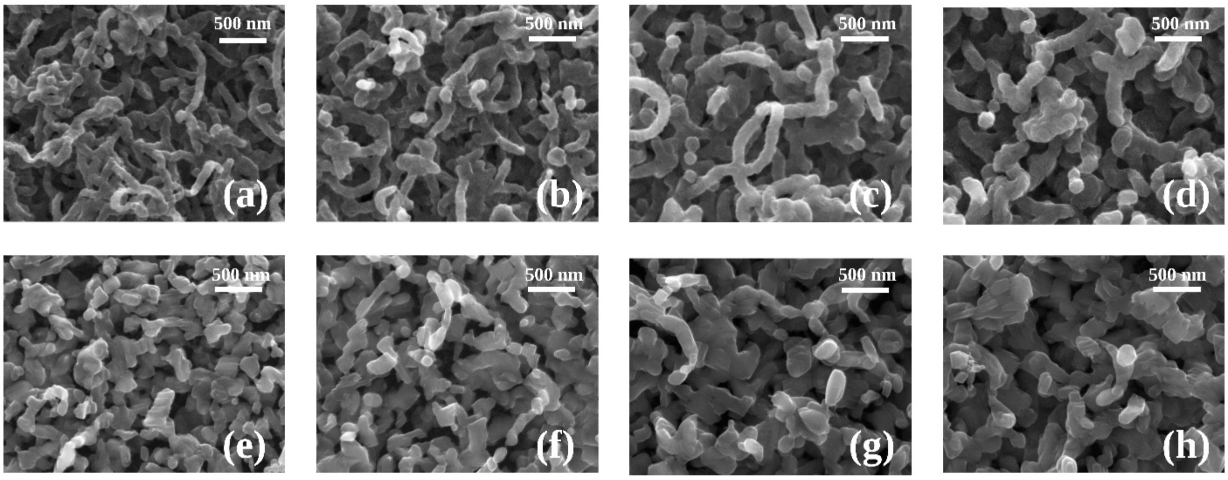

Figure S2. SEM images of oxidized samples with different vanadium depositions. At 400°C (a) 50 nm, (b) 100 nm, (c) 150 nm, and (d) 200 nm. At 500°C (e) 50 nm, (f) 100 nm, (g) 150 nm, and (h) 200 nm.

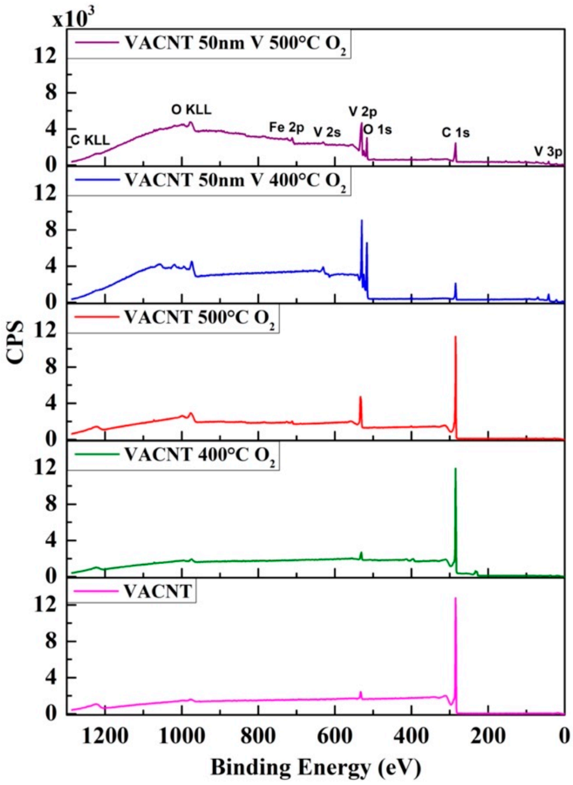

Figure S3. XPS survey of VACNTs as grown, VACNTs oxidized at 400 °C and 500 °C, and VACNT V_2O_5 samples with 50 nm of V thickness oxidized at 400 °C and 500 °C.
